# Perceived Barriers and Facilitators to Psychotherapy Utilisation and How They Relate to Patient’s Psychotherapeutic Goals

**DOI:** 10.3390/healthcare10112228

**Published:** 2022-11-07

**Authors:** Yvonne Schaffler, Thomas Probst, Andrea Jesser, Elke Humer, Christoph Pieh, Peter Stippl, Barbara Haid, Brigitte Schigl

**Affiliations:** 1 Department for Psychosomatic Medicine and Psychotherapy, University for Continuing Education Krems, 3500 Krems, Austria; 2 Austrian Federal Association for Psychotherapy, 1030 Vienna, Austria; 3 Department Psychology and Psychodynamics, Karl Landsteiner University of Health Sciences, 3500 Krems, Austria

**Keywords:** barriers, facilitators, psychotherapy, mental health services research, qualitative research, Austria

## Abstract

Access to psychotherapy is still limited by various barriers, and little is known about the facilitating circumstances. This study aims to assess self-reported barriers and facilitators to psychotherapy utilisation in private practice and how these access factors relate to psychotherapy goals as formulated by patients. The dataset consists of 21 face-to-face semi-structured interviews with patients treated by psychotherapists in private practice in Austria. Data were analysed using qualitative content analysis, including a frequency count of the number of codings to analyse relations between categories. A critical external barrier theme was unaffordable psychotherapy and confusion about how the Austrian funding system works. A negative experience with psychotherapy prior to the current one, such as not being understood and answered well enough by one’s therapist, was a frequently reported internal barrier. Individuals who faced more internal barriers and more external facilitators in seeking therapy, such as moral support from significant others and professionals, formulated less elaborate treatment goals. Although the study was carried out amid the COVID-19 pandemic, the pandemic played a minor role in patients’ self-reported barrier and facilitator themes.

## 1. Introduction

While a broad infrastructure for psychotherapeutic treatment exists in industrialised countries, access to psychotherapy is still limited by various barriers that preclude providing adequate services [1]. With the recent uptick in psychological distress around the globe since the COVID-19 pandemic [2], the demand for professional services increased even more [3]. However, not all individuals who would have profited from mental health care sought or found help. A prospective study on the predictors of health-seeking behaviour in Germany found that only 22.5% of persons with mental health problems sought support for them [4]. In Austria, little is known about the match between demand and supply. The outpatient psychotherapy demand is assumed to be significantly higher than the current treatment quota [5]. Psychotherapy utilisation in Austria is hampered by the many limitations and controls of financial coverage. Full reimbursement of psychotherapy visits through health insurance is far from meeting the needs of patients, varies depending on patients’ insurance provider and has waiting times of up to several months [6]. Other shortcomings in the effectiveness of care include the complex and bureaucratic application process [7], the continuing stigmatisation of mental health issues, information and communication deficits, and the lack of opportunities for psychosocially networked care in a closed care chain with psychiatric and psychosomatic services [8,9].

Knowing not only about perceived barriers but also about factors promoting positive attitudes and willingness to participate in psychotherapy can be influential in understanding mental health service use [9]. In general, people’s use of (mental) health services is a function of their predisposition to use services, the factors that enable or impede use, and their need for care [10]. The current research is a qualitative cross-sectional study, which describes the hindering and facilitating factors driving the utilisation of psychotherapy in Austria. The time frame of the study was from fall 2020 until fall 2021, in the midst of the COVID-19 pandemic. We know from previous studies that due to the pandemic, mental distress has also increased significantly in Austria [11,12].

Since psychotherapy goals are an essential factor related to clinical outcomes [13], and since goals might be influenced by determinants of access, a secondary aim is to explore how the barriers and facilitators to psychotherapy utilisation relate to therapeutic goals as patients formulate them.

### 1.1. Empirical Findings for Barriers and Facilitators

Barriers comprise internal (e.g., fear of being judged) and external (e.g., no funding for psychotherapy available) obstacles to psychotherapy utilisation. Facilitators may be internal (e.g., desire for a change) and external factors (e.g., family doctor’s advice to seek psychotherapeutic treatment) that aid in psychotherapy use. Perceived barriers and facilitators are usually assessed qualitatively by face-to-face interviews with open-ended questions [14,15,16]. In this way, patients can describe their experiences, reservations and preferences about using mental health services. Exploring the perceived barriers and facilitators to health care utilisation by qualitative methods provides additional and different information to quantitative survey questions about why people do or do not seek help [17].

Quantitative research suggests that the most critical external barriers to mental health service utilisation are structural and, as such, rooted in socio-demographic characteristics. They include the male gender, low levels of education and living in rural areas. Other critical but internal inhibiting factors are a higher level of psychopathology or a specific type of traumatic event [18]. Qualitative research also suggests that structural barriers are among the most frequently reported perceived external barriers. They include a lack of available mental health services and access problems due to language barriers or geographical distance. Further high-ranking perceived external barriers are time constraints, expenses and negative experiences with professional help. Frequently mentioned internal barriers are low mental health literacy (such as not recognising symptoms as mental health problems), stigma, shame or rejection, a lack of treatment-related knowledge or distrust and concerns about confidentiality [9]. Perceived facilitators lack attention in research. Out of 36 studies in a systematic review on perceived barriers and facilitators to mental health service utilisation in adult trauma survivors, only 8 addressed perceived facilitators, while all addressed perceived barriers. Out of those eight studies, seven were based on a qualitative design. Participants reported, for example, that they felt supported when peers or professionals encouraged them to seek professional help. Other facilitators addressed in qualitative studies were internal. The key internal facilitator themes were the severity of symptoms and the wish for a change that led to the decision to seek treatment. Other internal facilitators leading to mental health care utilisation were not wanting to burden the members of one’s family or experiences of trust, reduced stigma and social acceptance, as patients felt that their symptoms were understood and help was available [9,14]. An Austrian qualitative study including 21 adult participants over 23 years old who utilised either psychotherapy or rehabilitation or both found that bureaucratic obstacles (n = 9), stigmatisation (n = 7) and finding the right kind of help in the medical system (n = 6) were among the most frequently reported barriers. The most commonly reported facilitators were family members and friends who recommended initial treatment or psychotherapy to the person concerned (n = 7). Family doctors were among the most critical professionals who referred the patients to specialists, psychiatrists or psychotherapeutic treatment (n = 6) [6].

Individuals such as family members, friends, psychologists or doctors often support patients with mental health problems in their quest for therapy [19] because understanding how to handle difficulties is fundamentally based on social interaction [20]. However, it makes a difference whether the motivation to seek psychotherapy is more extrinsic or intrinsic when it comes to a patient’s motivation to engage with what a psychotherapist can offer. Research suggests that intrinsic pretreatment motivation is significantly associated with a patient’s active engagement in therapy to change and communicate [21]. Additionally, a significant relationship exists between a patient’s ratings of self-determined (vs. extrinsic) motivation and the intent to pursue therapy [22,23]. Poor motivation is a crucial, unspecific factor related to poor outcomes [24]. Could it therefore be that patients who enter mental health care only reluctantly and with great support from external facilitators are also reluctant to put great effort into defining the psychotherapeutic goals? Therapeutic goals are usually defined in collaboration between the patient and the psychotherapist. Active participation in goal setting mobilises patients to work towards these goals, thus increasing their motivation for treatment [25] and impacting the process and outcome [26]. The extent to which a goal is consciously articulated as opposed to being implicit in action at the beginning of psychotherapy defines how well a patient can set the agenda and orient their therapist to what needs to be done [27].

### 1.2. Aims and Research Questions

The primary aim of the current study is to advance insight into the process of access to psychotherapy by providing up-to-date qualitative knowledge regarding the perceived barriers and their facilitating counterparts in a group of adult patients since the autumn of 2020 amid the COVID-19 pandemic. The secondary aim is to find out how the reported “external barriers”, “internal barriers”, “external facilitators”, “internal facilitators” and “psychotherapeutic goals” are related. This leads to two research questions. Firstly, what barriers and facilitators hinder or facilitate psychotherapy utilisation in private practice? Secondly, are patients who formulate a high number of psychotherapeutic goals equally affected by barriers and facilitators as patients who formulate a low number of psychotherapeutic goals? Our findings should help researchers, gatekeepers and mental health professionals create strategies to enhance mental health care in times of increased demand. They should also shed light on how psychotherapists can harness what they know about their patients’ pathways to psychotherapy for setting therapy goals with their patients.

## 2. Materials and Methods

To achieve the goals of this paper, a qualitative descriptive design [28,29,30] was chosen, incorporating semi-structured individual interviews [31,32]. In healthcare research, qualitative descriptive designs are standard in fields with limited information on the subject [33]. They provide an insider view, seeking to replicate and characterise participants’ experiences [30]. We relied on content analysis to interpret the data and generate categories representing recurring features across participants’ accounts [34].

In a further step, we split the group of participants (N = 21). We undertook a comparison between a group that reported, relatively speaking, more psychotherapeutic goals compared to a group that reported, relatively speaking, fewer goals. We investigated which group mentioned a higher number of external barriers, internal barriers, external facilitators and internal facilitators to psychotherapy utilisation.

This study is part of a larger ongoing project on the Process and Outcome of Psychotherapy in Private Practice (POPP), which relies on an integration of quantitative and qualitative research methods. Individual psychotherapies are being researched under practical conditions in outpatient psychotherapeutic practices. In addition to the effectiveness (outcome), the process of psychotherapy is also examined, focusing on the therapeutic relationship as a process variable [35].

### 2.1. The Field of Research

The Austrian field of psychotherapy care offers patients many psychotherapeutic schools based on different theories about the development and course of disorders and corresponding emphasis in the treatment. Austria has 23 accredited psychotherapeutic schools [36] broadly categorised into four general theoretical orientations of thought: behavioural, humanistic, psychodynamic and systemic. Throughout all theoretical orientations, the individual patient’s goals of psychotherapy are not predetermined but discussed between the psychotherapist and the patient at the beginning of the treatment [37].

Psychotherapeutic services in Austria are mainly concentrated in cities and their surrounding areas. The proportion of psychotherapists working exclusively in private practice tends to increase and is currently around 63%. It can be assumed that there is an undersupply in some rural regions, as barriers, such as information deficits, social control and missing or distant offers, are more pronounced in remote rural areas [37]. While statutory health insurance fully covers acute psychiatric care, the reimbursement rates for outpatient psychotherapy in Austria are low. In most cases, the health insurance funds reimburse only part of the costs of the psychotherapy sessions. Private insurance covers costs to a greater extent but only for limited sessions. Fully financed psychotherapy is strongly contingent on governmental health insurance and therefore only available under certain conditions. In some areas, the initial contact with the system is regulated via so-called clearing centres. Trained therapists work in the clearing centres and advise patients about psychotherapy, the available therapeutic schools, local proximity, waiting time, group offers, etc. Particular attention should be paid to the urgency of treatment and social needs in each case. Special attention is paid to the care needs of children and adolescents, the elderly, addicts, disabled persons and persons suffering from psychosis. Other regions do not provide a specific point of contact at first access. The allocation of full reimbursement through insurance is up to some psychotherapists who have a contractual obligation to give preference, if possible, to patients with severe disorders and in socially difficult situations [38].

### 2.2. Recruitment

This study is based on data from patients treated by psychotherapists in private practice. The participating patients were invited by their psychotherapists to participate within a larger framework of the aforementioned study on the Process and Outcome of Psychotherapy in Private Practice (POPP) [35]. All psychotherapists participating in the POPP study were listed in the official list of licensed psychotherapists of the Austrian Federal Ministry for Social Affairs, Health Care and Consumer Protection. They provide individual outpatient psychotherapy in private practice. Recruitment started in the autumn of 2020. By the end of 2021, 90 therapists could be motivated to participate, and each psychotherapist was asked to invite 2–3 patients to also participate in the POPP study. The psychotherapists were asked to request those consecutive patients with whom psychotherapy was started without selection based on symptoms, symptom severity or personality traits to avoid bias.

Throughout 2021, a qualitative sub-study was conducted. All participating patients from the POPP study were asked to register actively for a qualitative interview via a homepage, and 24 patients took up the opportunity by June 2021. Ultimately, 3 patients withdrew their commitment or were unavailable, so we collected 21 interviews. Patients contacted us for participation in the qualitative sub-study from October 2020 up to June 2021. This study was conducted in accordance with the Declaration of Helsinki. Research ethical approval for the study was obtained from the Ethics Committee of Danube University Krems, Austria (EK GZ 28/2018-2021).

### 2.3. Data Collection

We chose semi-structured face-to-face interviews to yield the richness of data required in qualitative descriptive designs. The problem-centred interview enabled the interviewers to explore issues together with the participants by encouraging depth and rigour [31,32], thus facilitating the emergence of new concepts or topics [30]. Six researcher-trained psychotherapy students at the University of Continuing Education Krems (UWK) conducted and transcribed interviews with 21 patients during the summer/autumn of 2021. Intensive Zoom training on the interview procedure, access to the department’s scientific program, and occasionally, telephone contact and written correspondence between students and the qualitative research team of the POPP study (Y.S., A.J., B.S.), guaranteed interview quality.

The detailed and accurate interview guide used by the interviewers was designed for the POPP study to assess why patients decided to undergo psychotherapy; what goals and expectations they had in the beginning, how the psychotherapy developed; how they experienced their therapist as a person of the same or different gender; what aspects they found helpful and challenging in the psychotherapy process; if and how they perceived psychotherapy via phone or video conference during the lockdown, and what changed for them.

The participants chose the locations of the interviews, typically in their own homes, at a quiet café or at a psychotherapeutic practice. On average, the interviews lasted 67 min (ranging from 39 to 110 min). The interviews were recorded and then transcribed verbatim by the interviewers. The transcripts were given a study number and had all personally identifiable data removed, and transcripts were provided with a chiffre instead of the names of the participants.

### 2.4. Participants

The participants (N = 21) were patients who had sought help from a therapist in private practice between autumn 2020 and spring/summer 2021. Sixteen of their therapists were female. The number of sessions participants had attended with their current therapist when they were interviewed varied from n = 5 to n = 37, with an average of n = 20 sessions (see Table 1).

### 2.5. Data Analysis

#### 2.5.1. Content Analysis

A qualitative content analysis was used to analyse the obtained data [34] focusing on participants’ (N = 21) perceived barriers and facilitators to psychotherapy utilisation and on the therapeutic goals they had at the beginning of psychotherapy. We limited our analysis for this study part to passages in the interviews that followed the first narrative-generating questions and sub-questions from the POPP study’s interview guide, such as: “Can you please tell me why you decided to do psychotherapy?”; “Can you tell me about your life situation when you decided to seek treatment?”; “What do you remember about this time?”; “Were there people who supported your decision?”; “Can you tell me your expectations and goals when you started therapy?”. The interviewers allowed the participating patients to unfold their narratives, supporting them with immanent questions in a judgment-free atmosphere. Since the interviewees were granted a high degree of narrative freedom, they not only referred to the current reasons and pathways to psychotherapy utilisation but also their past experiences with the mental health care system.

**Figure 1:**
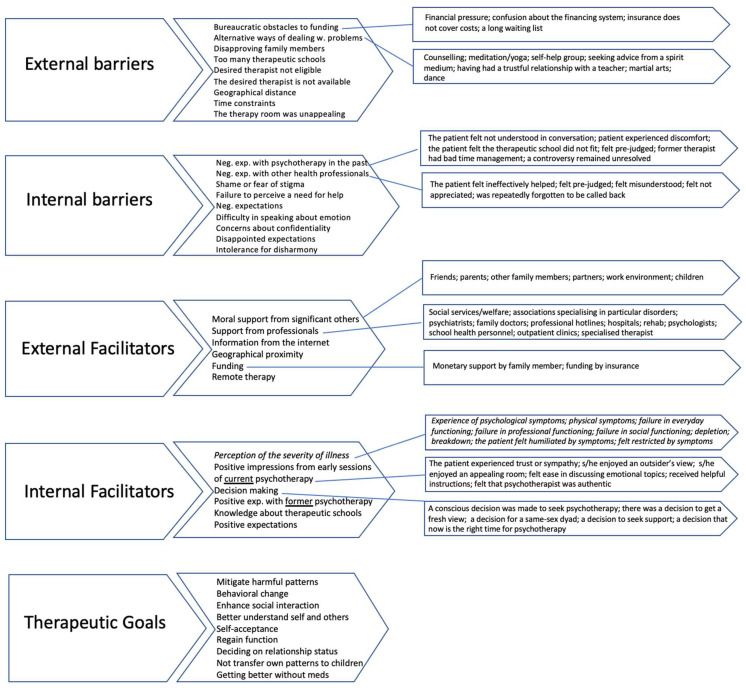
Code arrangements. First-order categories, second-order categories and third-order categories from left to right. In the internal facilitator category, need (but not enabling) characteristics are pointed out in italics.

After carefully reading the first interviews, the first author (Y.S.) coded all 21 interviews manually using ATLAS.ti Mac (Version 22.1.0) [39]. In line with the theoretical framework of Andersen’s behavioural model [10], a set of first-order categories (“external barriers”, “internal barriers”, “external facilitators”, “internal facilitators”) was a priori set to answer the first research question. Since Andersen’s model distinguishes between enabling and need characteristics, we used this distinction in the internal facilitator category (see Figure 1).

To answer our second research question, we also included an a priori set first-order category on goals (“psychotherapeutic goals”).

Each of the five first-order categories was then defined as a code group containing all the codings referring to second- and third-order categories. Common barriers and facilitators, such as “geographical distance” or “support from professionals”, were coded deductively based on a list of frequently found barriers and facilitators [9]. This list was supplemented by categories found inductively. Large deductively found second-order categories were split into inductively found third-order categories. Inductively found categories (as was the case, for example, with the many facets of decision making in the facilitator theme) were, conversely, also subsumed under a higher order category (for example, decision making) (see Figure 1).

Because this is a secondary analysis [40] of data that were collected to explore a broader set of questions, the answers of the study participants did not directly refer to barriers and facilitators to psychotherapy utilisation. They instead referred to positive or negative perceptions regarding psychotherapy in the past or present. These perceptions were then interpreted as either barriers or facilitators to utilising psychotherapy. For example, as far as positive expectations or thoughts regarding psychotherapy utilisation were uttered, they were framed as “internal facilitators.” In contrast, negative memories, perceptions or unfulfilled expectations were subsumed as “internal barriers”. One category, such as “negative expectations”, could be assigned (=coded) several times per case if several references to this category were made throughout a document (=interview transcript). The coding strategy and process were repeatedly discussed within the team of qualitative researchers (Y.S., A.J., B.S.). To answer the first research question and in preparation for answering the second research question, we described the content of the categories. In our description, the number of documents (one document = one case or participant) where the second- and third-order categories are grounded was cautiously considered an indicator of significance.

#### 2.5.2. Across-Group Comparison

**Figure 2:**
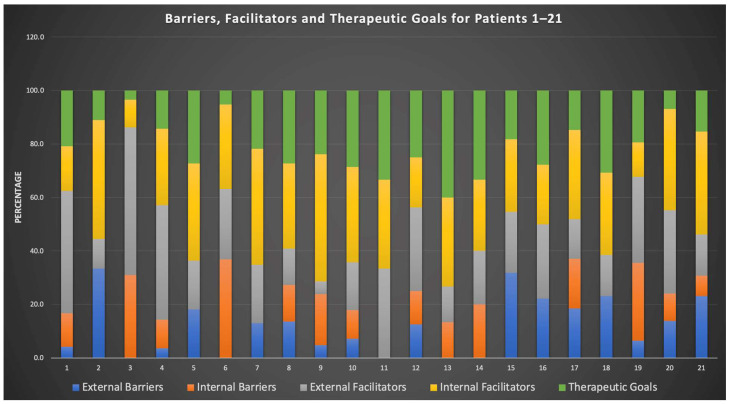
Relative distribution of external barriers, internal barriers, external facilitators, internal facilitators and psychotherapy goals for each case.

**Figure 3:**
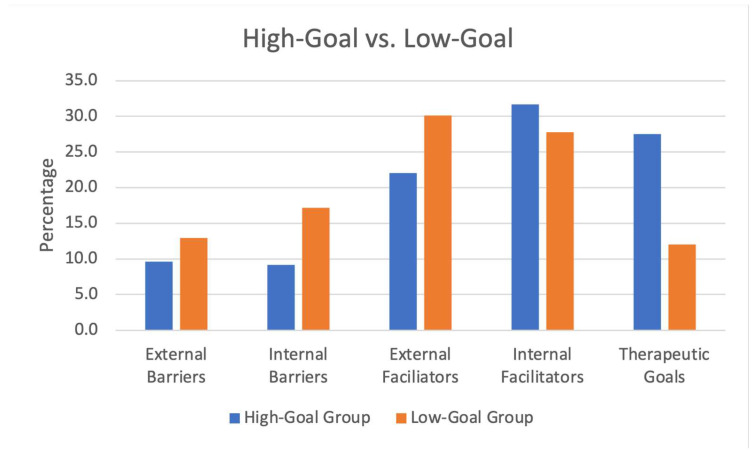
Relative distribution of external barriers, internal barriers, external facilitators, internal facilitators and psychotherapy goals for each case according to type. The high-goal type group consists of participants whose goals are >20% in relation to the other four items. The low-goal type group consists of participants whose goals are >20% in relation to the other four items.

To answer the second research question, we assumed that not all participants (N = 21) would equally elaborate on “external barriers”, “internal barriers”, “external facilitators”, “internal facilitators” and “therapeutic goals”. We therefore considered the frequency count of the number of codings, that is, the total of lower order categories belonging to each first-order category per case, as an indicator of significance. We proceeded in two steps. First, we determined the distribution of codings for the first-order categories per case. We used relative frequencies for comparing code distributions across the code groups (each first-order category is defined as a code group containing codings referring to second- and third-order categories), as percentages are easier to comprehend (see Figure 2). Second, we defined two types of cases based on the relative frequency of codings referring to “therapeutic goals”. We determined a cut-off score of 20%, along which the group was divided into approximately two halves. We divided the participants into a group with a high number of codings per first-order category “therapeutic goals” (>20%) and a group with a low number of codings per first-order category “therapeutic goals” (<20%) in relation to the number of codings per each of the other four first-order categories (see Figure 3). The Atlas.ti cross-tabulations underlying Figure 2 and Figure 3 provide more comprehensive information (see Supplement Table S1).

## 3. Results

### 3.1. Description of Categories

The categories are described as follows: Each first-order category forms a sub-section marked by a heading (e.g., “external barriers”). Within each of these sub-sections, the second-order categories are given, starting with the largest one. Second-order category numbers (referring to how many participants reported the respective category, n = X) are marked in bold. If a second-order category includes further sub-categories, these are reported, again, starting with the largest third-order category (n = X). Note that not all second-order categories are split into third-order categories, which is why not all bold second-order categories (n = X) are followed by third-order categories (n = X). Instead, a bold second-order category (n = X) might also be followed by another bold second-order category (n = X).

Selected statements from our participants are offered to illustrate how participants conceive of barriers, facilitators and goals.

#### 3.1.1. External Barriers

Participants named a variety of external barriers that prevented them from utilising psychotherapy. Bureaucratic obstacles to covering psychotherapy costs were cited most frequently (n = 11). Participants’ concerns about funding referred most often (n = 8) to financial pressure, which sometimes led to a limited frequency of sessions. One woman who was financially severely affected by the economic consequences of the COVID-19 pandemic reported her predicament between her need for psychotherapy and her need to economise as follows:

*(…) I thought, “I cannot afford this right now”. We [my husband and I] are self-employed, work in the arts and culture sector, and are massively affected by Covid. Moreover//um//I thought//um//the insurance pays a little extra, but it is marginal. So, therapy is something you must be able to afford (-)//be able//. So that was an issue. And I thought to myself, no, I cannot afford it now. And now I think, “yes, but I have to afford it” (female, age 45).*

Further concerns regarding therapy funding ranged from confusion about how the Austrian funding system worked (n = 3) to failed attempts to receive total funding (n = 2) due to long waiting lists, which discouraged participants from seeking total funding via governmental insurance (n = 1). The participants described the funding system as socially unjust, exhausting or pressuring.

Another frequently mentioned external barrier was that participants (n = 9) had intermittently found alternative ways to deal with their mental health problems rather than utilising psychotherapy. For example, three participants (n = 3) had undergone free counselling as a previous or interim solution before they took up psychotherapy (again), with one participant stating that men’s counselling had helped him overcome a tough time:

*How did I get to my new therapy now? Well, it was pretty tricky. In the end, I did not go for treatment for half a year because, at that time, I was feeling terrible, really//shit//[not good]. At this time, I went to men’s counselling for a while (male, age 28).*

Meditation and yoga were presented as other alternative practices that helped, as one participant had put it, “at a non-cognitive level” (n = 3). Other alternative healing contexts presented were visiting a self-help group (n = 1), getting advice from a spiritual healer (n = 1), having had a trustful relationship with one’s teacher (n = 1), having learned martial arts to defend oneself and getting to know one’s body (n = 1). Additionally, dance sessions were described as a supportive alternative context (n = 1):

*I said: hey, I need something that gives me pleasure. Something that invigorates. And I sort of started dancing hip-hop. (--) And after one and a half years of hip-hop, it became breaking. Well, that is usually called breakdancing. (--) And that is when I found myself again (male, age 39).*

Another reported external barrier was that close family members disapproved the participants seeing a psychotherapist (n = 4). For example, one female patient revealed that since she keeps visiting her psychotherapist, her husband…

*… feels that now a therapist is allying//herself with his wife against him (female, age 45).*

A further reported external barrier to participating in psychotherapeutic treatment was the variety of psychotherapeutic schools to choose from and the confusion that comes with trying to find the right one (n = 3). One patient reported that he initially felt so overwhelmed by the many options available in Austria that he was unable to decide on one therapist or school over an extended period:

*… I had an awful lot of first interviews, far too many, and I just did not manage to (-). I was completely overwhelmed with what was offered in Vienna because there were far too many therapists. I was utterly overwhelmed by the range of schools on offer because I did not know which type of therapy I should choose (male, age 28).*

Another patient further illustrated this point:

*(…) I find that//I find it somehow tricky. The search, and also finding someone. What criteria should be followed (female, age 34)?*

The other reported obstacles were that patients initially desired to work with a particular psychotherapist but found that they were not eligible due to a close personal or professional relationship with the patient (n = 2). A similar barrier reported was that patients desired to work with an expert from a specific school but found that this expert had no free capacity at the time they would have wanted to start their treatment (n = 2).

A different type of barrier to treatment utilisation was that patients lived too far away geographically from their psychotherapist to attend psychotherapy regularly (n = 2), that they had experienced time constraints, which prevented them from regularly attending because they were single parents (n = 1), and that they found the therapy room to be unappealing. Hence, they no longer wanted to go there (n = 1).

#### 3.1.2. Internal Barriers

The most frequently reported internal barrier was a negative experience with psychotherapy prior to the current one (n = 10). A negative former therapy experience was not having been understood and answered well enough (n = 7). The participants attempted to explain their former therapist’s unresponsive behaviour. For example, one patient tried to explain that a former therapist acted the way he did due to being overworked because of the pandemic:

*I also saw this therapist who just nodded the whole time, like “mhm,//mhm//mhm” I mean, he is not bad or anything. Still, the others also said that they did not like it, that he nods all the time, and so on.//Mhm//But I can understand it; it was just, he had a lot of work probably, a lot with Corona, too (female, age 20).*

In another case, a former therapist’s male gender was presumed to be why a female patient felt she could not talk openly. Another assumed cause of a failed therapeutic alliance was a misfit related to the therapist’s theoretical orientation (n = 3). According to one patient, this orientation was to blame for his emotional needs remaining unmet:

*The problem was that he (-) gave me the feeling that I was treating him instead of him treating me. So, he rambled a lot and had many theories; I could not do anything with that. I think that was because of his orientation; it did not fit (male, age 43).*

Further reported unsatisfactory former encounters with psychotherapists, some of whom were visited only once, included the experience of discomfort or relational distance (n = 3), feeling pre-judged (n = 2), experiencing that the session had ended before or after the regular end time (n = 1) and having unresolved controversy about a topic that felt important to the patient (n = 1). A different reported internal barrier was a negative experience with psychologists and doctors in general (n = 6). Participants felt that they were ineffectively helped (n = 3), given a diagnosis or judgement based on only superficial examination (n = 2), felt misunderstood (n = 1), felt not appreciated (n = 1) or were repeatedly forgotten to be called back (n = 1). Shame or fear of stigmatisation (n = 5) was a further reported internal barrier that caused participants to overthink before seeking psychotherapy:

*For us here, it [psychotherapy] is a somewhat notorious story. And I think almost no one in my circle has ever done one. Or just not told it, you know. It’s more of a secret discovery if someone finds out (laughs) (female, age 26).*

Some patients also stated that they hesitated because of a symptom-related failure to perceive a need to seek help, which they could not reflect upon at the given time (n = 4). Two patients reported failing to seek help in the past because of negative beliefs. They were convinced that psychotherapy could not help them (n = 2). Further internal barriers mentioned were that patients found it challenging to speak about emotional topics (n = 1), or that they felt concerned about the psychotherapist’s confidentiality (n = 1). Internal barriers that emerged during the participant’s current psychotherapy were unfulfilled expectations, e.g., that they missed concrete instructions or feedback from their therapists (n = 3). Another patient was afraid to communicate their genuine concerns or even prepared to quit therapy because of personal traits (n = 1):

*I am one of those people for whom everything has to fit. Otherwise, if there is disharmony, if something does not run smoothly, I end things or just tell them what they want to hear, and that is not the purpose of therapy.//mhm//(female, age 36).*

#### 3.1.3. External Facilitators

Mental health seeking also involves a social aspect. In this vein, all participants reported that they received moral support from significant others (N = 21) who recommended seeking psychotherapy and shared knowledge on finding a psychotherapist. Some recommended their own psychotherapist and passed on their contact details. Some took on an otherwise supportive role. This support mainly came from friends (n = 11). Consider the following statement about a friend who shared his own psychotherapy experiences with a participant in this study:

*And//mhm//I have a pretty good friend, yes, he is part of the family now, um, who regularly sees a therapist because he has epilepsy, and it is severe. And then we talked about it a bit. And that was then maybe all together, where I said, OK, now you tackle it and look for help.//mhm, mhm, yes//Yes (female, age 36).*

Additionally, worried parents (n = 10) and other family members, such as siblings and cousins (n = 10), helped the participants find treatment. Moral support through one’s partner (n = 7) and work environment (n = 3) was likewise reported:

*My boss is accommodating, his ears are open to such things, and then we said together that it would not be a mistake//ah//if I make sure that I get help (male, age 31).*

A further participant (n = 1) found herself in a situation where her adult daughters supported her health-seeking because they had noticed their mother’s struggle. External support for psychotherapy utilisation was not only provided by significant others but also by professionals (n = 11). These supporting professionals most often came from the clearing centres, social services or welfare organisations specialising in particular disorders. They provided help seekers with relevant information and a list of qualified therapists (n = 5). Additionally, psychiatrists encouraged psychotherapy utilisation for some participants (n = 5), followed by family doctors (n = 4) and professional helplines (n = 3). One participant revealed that he was counselled on where to seek help in rather specific terms through a helpline:

*Um, the hospital was not open or did not pick up the phone, and then I called the//ahm//the telephone, the telephone helpline (-). And a trauma therapist picked up the phone. And I told her in a few minutes what was going on with me. And she said that I had emotional childhood trauma. She recognised it quickly and told me to seek body-oriented individual therapy (male, age 30).*

Other healthcare providers supporting this study’s participants in seeking psychotherapy in private practice worked in hospitals (n = 2), psychiatric rehabilitation clinics (n = 2), or worked as clinical counsellors (n = 2), school physicians or school psychologists (n = 2). Additionally, an outpatient clinic (n = 1) was mentioned. One participant said that she was referred to individual psychotherapy by her and her partner’s couple therapist (n = 1). Another participant was recommended to undergo psychotherapy by her child’s therapist, who suggested that parents of troubled children should also seek treatment (n = 1). Information about psychotherapy utilisation was sometimes also found on the internet, which participants (n = 6) described as a source of valuable information regarding therapeutic schools, practice locations and photographs of psychotherapists. Psychotherapists’ professional homepages were described as providing relevant information to making a good match with a therapist:

*Well, how I chose her was simple, I saw a picture of her, and I liked that. And her homepage was appealing. And with the others, I felt more like//ahm//I did not know if they would suit me because they were for older people, not younger people. And with her, I immediately felt that (-) we would fit (female, age 29).*

Other external factors that facilitated the use of psychotherapy were the physical proximity to a psychotherapist’s practice, so as not to travel long distances (n = 3). Living close to a psychotherapeutic practice was not only seen as beneficial for quick access but also for better mutual understanding:

*I think it is essential that a therapist comes from the same region. Because she [the psychotherapist] has the same//um//background, she has grown up in the same circumstances//living the same village life (female, age 26).*

As we already know from the external barrier section, the way to finance psychotherapy is crucial to accessing it (n = 6). In this vein, three participants reported receiving full or partial financial support from their parents or other family members (n = 3). Another group of three felt relieved because they received full or partial funding from their insurance (n = 3). One participant expressed alleviation at having found Skype as a medium of communication with the therapist, as it is more flexible and saves long journeys (n = 1):

*We do it like this, um, I can do it via Skype,//aha//which helps me terribly because it’s a twenty-kilometre drive into the city. And with the children and my husband working shifts and then a shared car,//mhm, mhm//And she [the psychotherapist] only has certain days she is available, which narrows it down a lot. (…) It is very convenient for me that I can do it via Skype. And, um, I think that also helped with Corona greatly (female, age 36).*

#### 3.1.4. Internal Facilitators

More than half of the participants (n = 16) stated that they experienced a peak or crisis point in their suffering, which led to a desire for change, and thus, to seeking treatment. Fourteen (n = 14) of them used psychological vocabulary to describe the experienced phenomena. They talked about anxiety, panic attacks, depressive moods, self-mutilation or suicidal thoughts. The experience of physical symptoms (n = 4), a failure to cope with everyday life (n = 4), a loss in professional activity (n = 4), exhaustion (n = 2), a breakdown experience (n = 2), feeling humiliated (n = 1) or limited by one’s symptoms (n = 1) were other acute problems formulated in more everyday language, which contributed to making participants feel uncomfortable enough to seek treatment.

After finding their current psychotherapist, most participants (n = 16) reported having had a positive impression from their early sessions. Nine (n = 9) of them said they immediately felt trust or sympathy for their therapist. One participant illustrated this point through a revealing example in which she was encouraged to tell if something felt wrong immediately:

*It impressed me that she said: You do not have to stay here [in psychotherapy] only to visit. You may say: this does not fit me. Alternatively, you can always say it does not work for me anymore. So, I felt I could tell her that at any time. (-) And that//I think//already inspired much trust in me when I first saw her (female, age 26).*

Participants also reported that it felt good to get an unbiased perspective on one’s problems (n = 5), that they found the therapy room atmospherically and aesthetically appealing (n = 5), that they found it easy to talk about emotional issues (n = 2), that they received helpful instructions right away (n = 1) and that they liked that their psychotherapist seemed to be an authentic person (n = 1). Another large category was “decision making,” endorsed by fifteen participants (n = 15). Eight participants (n = 8) stated that they had taken a conscious decision to seek treatment even though they were not necessarily alone in the decision-making process. Others (n = 5) had decided to look for a new psychotherapist because they wanted a new perspective on their problems. Working in a same-sex dyad was an operational decision for some female participants (n = 5) who wanted to discuss gender-specific aspects with a female psychotherapist:

*There are things I only want to discuss with a woman.//mhm, mhm//where I certainly do not need a man’s point of view (female, age 48).*

Five participants reported a general desire for support (n = 5). Three participants (n = 3) stated that their recent decision to seek treatment had to do with feeling that now was “the right time” to reflect. One participant used the metaphor of “tidying up from the inside” to illustrate her point:

*I remember that last autumn, there was this lockdown, and I had the feeling that now we have, I do not know, two or three weeks where you can reflect on yourself (-). So you use this dark time of year, my birthday, it is always like that, and//ahm//to reflect, look at things that you do not usually get to, and tidy up from the inside. And that started this process (female, age 48).*

Prior psychotherapy utilisation, as long as it was mentioned in a neutral or positive light, was likewise interpreted as a facilitator to current psychotherapy utilisation. Twelve participants reported having utilised psychotherapy before their current therapy (n = 12), which they had found helpful…

*Yeah, so, having done therapy before in 2019, I have known that it helps me (female, age 22).*

Knowledge about psychotherapy approaches and personal needs was another large category of internal facilitators to participation in psychotherapy. About half of the patients (n = 10) reported knowledge or at least some ideas about the therapeutic school they were about to choose and why a particular approach with a particular set of methods would suit their needs:

*Once I came to her, my priorities at that time were already bodywork and feeling understood; those were priorities number one (male, age 28).*

A third of the participants (n = 7) stated they had general positive expectations and hoped psychotherapy could help them:

*I have never//wished//[to be in psychotherapy] as much as I did back then because I just knew it would help me (male, age 28).*

#### 3.1.5. Patients’ Psychotherapeutic Goals

All participants (N = 21) could describe at least one goal-related aspect they wanted to achieve with the help of psychotherapy. Almost half of them recognised and reported harmful patterns of emotion, thoughts or behaviours they wanted to alter (n = 10), such as:

*I wanted to learn ways to work on specific memories, associations or experiences. They were always there anyway, but I never actively worked on them. It always felt very stagnant, and with psychotherapy, I learned to bring things into a flow (male, age 28).*

Or, as another participant put it:

*I want to be able to go back to work and learn from my mistakes so that I do not slip into the old patterns again (female, age 37).*

The more unidimensional goals mentioned were to change one’s behaviour (n = 8), to improve one’s social interactions (n = 8), to understand oneself and others better (n = 6), to find joy in life again (n = 6), to clarify one’s professional goal (n = 5), to strengthen one’s self-worth (n = 5), to take control of one’s own life, instead of just pleasing others (n = 5), to accept oneself and one’s circumstances (n = 4), to function again in everyday life and at work (n = 3), to decide on a relationship status (n = 2), not to transfer one’s old patterns to one’s children (n = 2) and to become better without pills (n = 1).

### 3.2. Distribution of Barriers, Facilitators and Psychotherapeutic Goals

#### 3.2.1. Distribution per Case

Across cases, the codings referring to the five items were distributed as follows: external barriers (n = 49 or 11.3%, ranging from 0 to 7 codings per case), internal barriers (n = 57 or 13.1%, ranging from 0 to 9 codings per case), external facilitators (n = 113 or 26%, ranging from 1 to 16 codings per case), internal facilitators (n = 129 or 29.7%, ranging from 3 to 10 codings per case) and psychotherapy goals (n = 86 or 19,8%, ranging from 1 to 8 codings per case). See Figure 2 for visualisation. For more details, see the Cross-Table S1 in the Supplement.

#### 3.2.2. Distribution per Case in Two Groups

Using a cut-off score of 20% to divide the participants into a group with a high number of codings per item and a group with a low number of codings per item, we defined a high-goal group (>20% of codings for item “therapeutic goals”, n = 12) and a low-goal group (<20% of codings for item “therapeutic goals”, n = 9).

Across cases in the high-goal type group, the codings referring to the five first-order categories were distributed as follows: external barriers (N = 21 or 9.7%, ranging from 0 to 4 codings per case), internal barriers (n = 20 or 9.2%, ranging from 0 to 4 codings per case), external facilitators (n = 48 or 22%, ranging from 1 to 11 codings per case), internal facilitators (n = 69 or 31.7%, ranging from 3 to 10 codings per case) and psychotherapy goals (n = 60 or 27.5%, ranging from 1 to 8 codings per case). For more details, see the Cross-Table S2 in the Supplement.

Across cases in the low-goal type group, the codings referring to the five first-order categories were distributed as follows: external barriers (n = 28 or 13%, ranging from 0 to 7 codings per case), internal barriers (n = 37 or 17.1%, ranging from 0 to 9 codings per case), external facilitators (n = 65 or 31.1%, ranging from 2 to 16 codings per case), internal facilitators (n = 60 or 27.8%, ranging from 3 to 11 codings per case) and psychotherapy goals (n = 26 or 12%, ranging from 1 to 6 codings per case). For more details, see the Cross-Table S3 in the Supplement.

The distribution of codings referring to the five first-order categories in the high-goal and the low-goal group indicates that patients with a high number of goals mentioned fewer external barriers to treatment, fewer internal barriers to treatment, fewer external facilitators and more internal facilitators to treatment (see Figure 3). For more details, see visualisation A4 and the Cross-Table S5 in the Supplement.

## 4. Discussion

The present study sought to investigate the perceived barriers and facilitators to psychotherapy utilisation (research question 1), their relation to psychotherapy goals as patients formulated them at the beginning of their treatment and whether patients who formulated a high number of psychotherapeutic goals were equally affected by barriers and facilitators as patients who formulated a low number of psychotherapeutic goals (research question 2).

To better understand the results presented, it helps to reiterate the central features of the psychotherapeutic system in Austria. A variety of 23 psychotherapeutic schools are practised in Austria, for example, systemic therapy, integrative psychotherapy, concentrative movement therapy, logotherapy or gestalt-theoretical psychotherapy, each of which belongs to one of four general theoretical orientations. Then, only limited places for fully funded psychotherapy are available, and there is no national uniform strategy to inform patients about their options for funding. Indeed, the characteristics of our sample suggest that only four (n = 4) out of all (N = 21) participants received total funding, with the majority (n = 15) being only partially reimbursed.

Yet another particularity should be kept in mind when discussing this paper’s results: the pandemic situation at the time of data collection.

### 4.1. Discussion of Categories

#### 4.1.1. External Barriers

The results show that one central external barrier theme in facilitating psychotherapy is that of affordable and accessible psychotherapy. Half of this study’s participants (n = 11) mentioned obstacles to covering psychotherapy costs, which caused distress through financial pressure in 8 (n = 8) participants. The rest (n = 3) reported confusion about how the system worked, failed attempts to receive total funding and long waiting times. Other qualitative studies, too, have identified such barriers for patients in Austria [6,14]. Austrian psychotherapists also reported difficulties with the bureaucratic application process [7]. Studies have shown that the lack of coverage of treatment costs is a significant barrier preventing patients from entering psychotherapeutic treatment [9]. Providing coverage for psychotherapy and for mental health services, on the other hand, would be a cost-efficient investment in the short and the long term [41].

Since this study was conducted during the COVID-19 pandemic, during which the rates of depression, anxiety, insomnia and stress have increased 2–8 times among the general population [42,43,44], and since the pandemic caused socio-economic disruption on a global scale [45], we assume that problems in accessing affordable psychotherapy hit individuals suffering from mental health problems even harder than before.

Almost half of our sample (n = 9) sought alternative ways of dealing with their mental health problems, such as counselling, yoga, meditation, seeking a spiritual medium, etc., before they started seeing their current psychotherapist. While this is not unusual, it may well be related to the fact that these alternatives were cheaper and more accessible. We would like to clarify that we understand these alternatives only in the sense of a barrier in that they may have delayed the start of therapy or that they were seen as a temporary replacement for it. Therefore, the question remains as to whether the participants would have profited more if they had had better access to psychotherapy and sought treatment earlier.

#### 4.1.2. Internal Barriers

A widespread internal barrier theme is that of a previous negative psychotherapy experience. Almost half (n = 10) of the interviewed patients stated that they felt misunderstood or ignored regarding their core concerns in earlier psychotherapy, that is, before seeing their current psychotherapist. This underlines the importance of therapists providing their patients with contextualised and individualised conceptualisations of their problems right at the beginning of treatment. Not paying attention to some core aspects of the patient’s difficulties, as experienced by the patient themselves, can be a significant obstacle to future therapeutic work [46]. Experienced psychotherapists therefore ensure that what happens in therapy is meaningful for the patient and relevant to the attainment of the patient’s goals and are aware of the complexity of the process, e.g., the patient’s ambivalence and resistance to change [47]. In three cases, dissatisfaction with one’s psychotherapist also referred to the patient’s current psychotherapy. These patients (n = 3) said they missed concrete instructions or feedback from their psychotherapists. This resonates with a qualitative study based on interviews with dissatisfied patients in Sweden, which suggested that unhappy patients wanted more responses from their therapists [48].

A quarter of our sample patients (n = 5) reported shame or fear of stigmatisation, which underlines the need for improvement in the de-stigmatisation of psychotherapeutic treatment, as pointed to also by the respondents of another Austrian study [6].

#### 4.1.3. External Facilitators

The importance of social support in seeking professional help [19] was reflected in that all participants (N = 21) reported receiving moral support from significant others. As has been described by survivors of child abuse in a recent Austrian study on barriers and facilitators [14], the participants of this study also reported that their friends, family and other companions did not only recommend psychotherapy in general. Instead, they shared their psychotherapy experiences, named specific recommendations and gave the participants contact details of psychotherapists they trusted.

On the other hand, the professionals only supported half of this study’s participants (n = 11). Additionally, their recommendations were often very concrete. For example, they provided the participants with lists of qualified therapists or recommended certain types of therapy for specific disorders. These results suggest that missing centralised information through nationwide campaigns is compensated through support from the patients’ friends and families and, to a lesser extent, also through help from professionals.

The more self-directed way in which patients acquire relevant information is through the internet. This study’s participants (n = 6) reported having found homepages in which psychotherapists present themselves via photographs, personal information and the specifics of their therapeutic school.

Regarding the coverage of psychotherapy costs, six (n = 6) participants mentioned that they received funding through their relatives or insurance, which they found was essential support for accessing psychotherapy. This again highlights that the financial means or insurance coverage for mental health service use is paramount to enabling psychotherapy [49].

#### 4.1.4. Internal Facilitators

More than two-thirds of our participants reported a high level of psychological distress and symptom severity as a crucial need factor [10] for engaging in psychotherapy (n = 16). This is consistent with prior findings of key facilitators associated with treatment seeking [9,18] and with findings from a quantitative survey in which self-reported distress was significantly associated with deciding that therapy might help and with seeking therapy [50]. Realising that there was a problem has been described as the most challenging step in the process of seeking therapy [51]. Our results also indicate that fourteen participants (n = 14) applied psychological terms to describe their experiences of suffering, suggesting that they had already internalised psychological concepts. This appears to be connected to the rich experience that the patients brought with them when this study was conducted, as half (n = 12) of them stated that they had been in treatment for some time before their current psychotherapy. This result also suggests that having seen a psychotherapist in the past makes an individual likely to seek psychotherapy again, even if not only positive memories are associated with the previous treatment.

The other categories refer to enabling characteristics [10], that is, to inner resources or ego functions of the participants, such as the ability to build relationships, make decisions or acquire knowledge. In this vein, more than two-thirds of this study’s participants (n = 16) reported having had a positive impression from their early sessions with feelings of trust or sympathy. This points towards a constructive future development of the running psychotherapeutic processes because trust and sympathy are a good basis for therapeutic alliance. The quality of the alliance is a robust predictor of therapy outcome. The relationship between the alliance and the eventual therapeutic outcome is quite apparent as early as in the third session of treatment [52,53]. Among the reported reasons for the positive impressions from early sessions were that patients felt encouraged to speak out, that they felt comfortable and that they experienced their therapist as authentic. This is important, as therapists should encourage their patients to report unwanted effects back to their therapists [54].

Another large internal facilitator category (n = 15) regarded different aspects of active decision making. The decisions remembered and reported by the participants referred to different areas, such as having taken a conscious decision to seek treatment (n = 8), having decided to get a fresh perspective on old problems (n = 5), having decided to work in a same-sex dyad (n = 5) or having felt that the “right time” has come for treatment (n = 3). We think these reported moments of decision-making point towards an intrinsic pretreatment motivation, which is significantly associated with a patient’s active engagement in therapy to change and communicate [21]. The reported decision to work in a same-sex dyad (n = 5) also speaks to the empowerment of women who are aware of possible harmful gender dynamics. Their concern is backed by gender research in psychotherapy, showing that specific issues are more challenging to deal with in a male therapist–female patient gender constellation [55,56].

Considerable mental health and psychotherapy literacy among this study’s participants is demonstrated by the fact that half of them had already thought about which school of therapy to choose (n = 10). This relates to the high rate of treatment experience in this sample and the fact that the Austrian therapy system, with its variety of schools, suggests and inspires such considerations.

A third (n = 7) of the participants uttered that they had hope or positive expectations towards psychotherapy, which, according to the literature, significantly predicts the rates of improvement [57,58].

Again, also in this section, the theme of psychotherapeutic schools was recurrent, as it appeared in the patients’ considerations for planning psychotherapy.

#### 4.1.5. Psychotherapeutic Goals

The goals described by this study’s participants are manifold, and all participants could at least describe one aim they wanted to achieve. In general, the formulation of goals results from a collaboration between the patient and the psychotherapist, and there is research evidence that this collaboration is related to client outcomes [26]. Except for two goals, namely not to transfer one’s old patterns to one’s children (n = 2) and to become better without pills (n = 1), which may be classified as avoidance goals, all reported goals were approach goals. Framing goals using avoidance terms was shown to be associated with less symptomatic improvement but did not affect goal attainment [59].

### 4.2. Discussion of Distribution of Barriers, Facilitators and Psychotherapeutic Goals

Comparing the distribution of the previously discussed categories on a case-by-case basis (Figure 2), a heterogeneous pattern emerged, showing that some participants did not report external barriers (cases 3, 6, 13, 14). In contrast, others did not report internal barriers (cases 2, 5, 7, 11, 15, 16). On the other hand, all participants have reported external and internal facilitators and at least one goal. When looking at cases with disproportionately high internal barriers (cases 3 and 6), it becomes apparent that these participants have reported relatively few aims. This could hint at an answer to the second research question, suggesting that participants who formulated a low number of psychotherapeutic goals showed a more barrier- and external-facilitator-driven pattern than patients who formulated a high number of psychotherapeutic goals.

Comparing two sub-groups, one group is defined as a high-goal type group (>20% goals in relation to the other first-order categories). The other group, as a low-goal type group (<20% goals in relation to the other first-order categories) (Figure 3), suggests that patients from the low-goal group indeed reported more external barriers to treatment and more internal barriers to treatment. Moreover, the low-goal group also appears to be driven by external rather than internal facilitators, which suggests more extrinsic than intrinsic motivation to seek treatment. This, in turn, aligns with the fact that more externally motivated patients engage less actively in therapy [21], which makes them less likely to collaborate on formulating therapy goals with their psychotherapist.

### 4.3. Limitations and Strengths

This study has several limitations. First, we cannot rule out a possible selection bias, as participants willing to participate in the POPP study’s qualitative sub-study may have been more likely to open up and seek professional help. Second, there is also a potential for recall bias. Since this study’s participants saw their current psychotherapists already for n = 20 sessions, on average, at the time they were being interviewed, it cannot be excluded that their recent experiences with psychotherapy did alter the memories of reported psychotherapy processes from the past. Third, regarding data analysis, data were evaluated and interpreted outside their immediate survey context and set into the theoretical context of health-seeking behaviour. Therefore, our analysis is a secondary analysis, meaning that data were taken out of their initial narrative form and re-contextualised [40] into the framework of barriers and facilitators [9]. Fourth, the frequent occurrence of categories was taken as an indicator of greater importance, but it might also simply reflect a greater willingness or ability to discuss a topic [60]. Fifth, regarding the internal barrier categories “negative experience with psychotherapy” and “negative experience with other mental health professions”, we did our best to distinguish between the two. However, due to the potential for confusion between the mental health professional strands among patients, it could be that no clean separation has taken place here. Sixth, the result in response to the second research question is preliminarily due to the low number of cases and the indirect way in which patients were asked about barriers and facilitators.

Nevertheless, the emerging hypothesis seems plausible enough to be tested with a larger sample and an interview guide that includes specific questions about barriers. For future research, we recommend a larger sample and the inclusion of predisposing characteristics, such as demographic characteristics, particularly age and gender, and social structure, as proposed by Andersen’s behavioural model [10]. Moreover, the classification of barriers and facilitators does not always allow a clear assignment of the sub-categories. For example, self-care practices, such as meditation and yoga, can be read as both barriers to psychotherapy utilisation and enabling facilitators. Another problem with the existing categorisation is that the inner facilitator category consists of both need and enabling characteristics, which have distinct qualities. We, therefore, propose to look for correlations among themes (also) without dichotomous classification into barriers and facilitators.

A strength of this study is that extensive interviews were conducted face-to-face in an open and safe atmosphere, examining the participants’ individual experiences in their own context. As the participants were allowed to tell their own stories with psychotherapy, the facilitators, a concept on which there has been little research so far [9], could be explored in depth.

## 5. Conclusions

Although COVID-19 increased the demand for mental health services, the pandemic played a minor role in patients’ self-reported barrier and facilitator themes. Since a critical external barrier theme was unaffordable psychotherapy and confusion about how the Austrian funding system works, reducing this barrier would mean providing better access to mental health services, including a nationwide information campaign, an increase in insurance subsidies and more fully refunded psychotherapy. Our findings also have implications for clinical assessment. This study’s participants were less likely to formulate elaborate treatment goals when they had greater internal barriers and more external support in seeking treatment. Thus, psychotherapists should particularly emphasise working with their patients to develop psychotherapy goals when they know that their patients have been sent to therapy or when they report several internal barriers.

## Figures and Tables

**Table 1:** Sample characteristics (N = 21).

	N	%
**Gender**		
Female	16	76.2
Male	5	23.8
**Age in years**		
18–29	9	42.9
30–39	7	33.3
40–49	4	19.0
50–59	1	4.8
**Highest level of education**		
Secondary school	4	19.0
Apprenticeship	1	4.8
High school	10	47.6
University	6	28.6
**Residence**		
≤5000 inhabitants	5	23.8
≥5000/≤25,000 inhabitants	5	23.8
≥25,000 inhabitants	11	52.4
**Therapy costs**		
Fully funded by insurance	4	19.0
Fully privately funded	2	9.5
Partial reimbursement through insurance	15	71.4
**Orientation**		
Psychodynamic	7	33.3
Humanistic	10	47.6
Systemic	3	14.3
Behavioural	1	4.8

## Data Availability

Data are available upon reasonable request.

## Supplementary Materials

The following supporting information can be downloaded at: https://www.mdpi.com/article/10.3390/healthcare10112228/s1, Supplement to Section S3.2.1: Cross-table S1.; Supplement to Section S3.2.2: Cross-tables S2 and S3, Visualisation S4 and Cross-table S5.
