# Supplementary material for: Perceived Barriers and Facilitators to Psychotherapy Utilisation and How They Relate to Patient’s Psychotherapeutic Goals
Source: Healthcare (Basel). 2022 Nov 7;10(11):2228. doi: 10.3390/healthcare10112228 (PMC9690172; doi:10.3390/healthcare10112228)
Supplement: Supplementary file 1 [file healthcare-10-02228-s001.zip › healthcare-2012307-supplementary.pdf]

Supplement to 3.2.

3.2.1.

Case	Quotations	3_External Barrie... (◇ 20, quotations 49)	4_Internal Barrier... (◇ 20, quotations 57)	5_External Facilit... (◇ 23, quotations 113)	6_Internal Facilit... (◇ 24, quotations 129)	7_Goals (◇ 13, quotations 86)	Totals
1	46	1<br>4.17 %	3<br>12.50 %	11<br>45.83 %	4<br>16.67 %	5<br>20.83 %	24<br>100 %
2	39	6<br>33.33 %		2<br>11.11 %	8<br>44.44 %	2<br>11.11 %	18<br>100 %
3	55		9<br>31.03 %	16<br>55.17 %	3<br>10.34 %	1<br>3.45 %	29<br>100 %
4	55	1<br>3.57 %	3<br>10.71 %	12<br>42.86 %	8<br>28.57 %	4<br>14.29 %	28<br>100 %
5	27	2<br>18.18 %		2<br>18.18 %	4<br>36.36 %	3<br>27.27 %	11<br>100 %
6	37		7<br>36.84 %	5<br>26.32 %	6<br>31.58 %	1<br>5.26 %	19<br>100 %
7	41	3<br>13.04 %		5<br>21.74 %	10<br>43.48 %	5<br>21.74 %	23<br>100 %
8	42	3<br>13.64 %	3<br>13.64 %	3<br>13.64 %	7<br>31.82 %	6<br>27.27 %	22<br>100 %
9	40	1<br>4.76 %	4<br>19.05 %	1<br>4.76 %	10<br>47.62 %	5<br>23.81 %	21<br>100 %
10	47	2<br>7.14 %	3<br>10.71 %	5<br>17.86 %	10<br>35.71 %	8<br>28.57 %	28<br>100 %
11	30			4<br>33.33 %	4<br>33.33 %	4<br>33.33 %	12<br>100 %
12	34	2<br>12.50 %	2<br>12.50 %	5<br>31.25 %	3<br>18.75 %	4<br>25.00 %	16<br>100 %
13	37		2<br>13.33 %	2<br>13.33 %	5<br>33.33 %	6<br>40.00 %	15<br>100 %
14	36		3<br>20.00 %	3<br>20.00 %	4<br>26.67 %	5<br>33.33 %	15<br>100 %
15	42	7<br>31.82 %		5<br>22.73 %	6<br>27.27 %	4<br>18.18 %	22<br>100 %
16	38	4<br>22.22 %		5<br>27.78 %	4<br>22.22 %	5<br>27.78 %	18<br>100 %
17	51	5<br>18.52 %	5<br>18.52 %	4<br>14.81 %	9<br>33.33 %	4<br>14.81 %	27<br>100 %
18	31	3<br>23.08 %		2<br>15.38 %	4<br>30.77 %	4<br>30.77 %	13<br>100 %
19	50	2<br>6.45 %	9<br>29.03 %	10<br>32.26 %	4<br>12.90 %	6<br>19.35 %	31<br>100 %
20	48	4<br>13.79 %	3<br>10.34 %	9<br>31.03 %	11<br>37.93 %	2<br>6.90 %	29<br>100 %
21	37	3<br>23.08 %	1<br>7.69 %	2<br>15.38 %	5<br>38.46 %	2<br>15.38 %	13<br>100 %
		49<br>11.29 %	57<br>13.13 %	113<br>26.04 %	129<br>29.72 %	86<br>19.82 %	434<br>100 %

☐ = number of cases ◇ = number of codes (quotation icon) = number of coded quotations

Cross-Table S1. underlying the Distribution of Barriers, Facilitators and Therapeutic Goals per Case

3.2.2.

Case	Quotations	3_External Barrie... (codes 20, quotations 49)	4_Internal Barrier... (codes 20, quotations 57)	5_External Facilit... (codes 23, quotations 113)	6_Internal Facilit... (codes 24, quotations 129)	7_Goals (codes 13, quotations 86)	Totals
1	46	1<br>4.17 %	3<br>12.50 %	11<br>45.83 %	4<br>16.67 %	5<br>20.83 %	24<br>100 %
5	27	2<br>18.18 %		2<br>18.18 %	4<br>36.36 %	3<br>27.27 %	11<br>100 %
7	41	3<br>13.04 %		5<br>21.74 %	10<br>43.48 %	5<br>21.74 %	23<br>100 %
8	42	3<br>13.64 %	3<br>13.64 %	3<br>13.64 %	7<br>31.82 %	6<br>27.27 %	22<br>100 %
9	40	1<br>4.76 %	4<br>19.05 %	1<br>4.76 %	10<br>47.62 %	5<br>23.81 %	21<br>100 %
10	47	2<br>7.14 %	3<br>10.71 %	5<br>17.86 %	10<br>35.71 %	8<br>28.57 %	28<br>100 %
11	30			4<br>33.33 %	4<br>33.33 %	4<br>33.33 %	12<br>100 %
12	34	2<br>12.50 %	2<br>12.50 %	5<br>31.25 %	3<br>18.75 %	4<br>25.00 %	16<br>100 %
13	37		2<br>13.33 %	2<br>13.33 %	5<br>33.33 %	6<br>40.00 %	15<br>100 %
14	36		3<br>20.00 %	3<br>20.00 %	4<br>26.67 %	5<br>33.33 %	15<br>100 %
16	38	4<br>22.22 %		5<br>27.78 %	4<br>22.22 %	5<br>27.78 %	18<br>100 %
18	31	3<br>23.08 %		2<br>15.38 %	4<br>30.77 %	4<br>30.77 %	13<br>100 %
		21<br>[illegible]	20<br>[illegible]	48<br>22.02 %	69<br>31.65 %	60<br>27.52 %	218<br>100 %

3_External Barriers to Treatment

= number of cases = number of codes = number of coded quotations

Cross-Table S2. underlying the High-Goal Type Cases

Case	Quotations	3_External Barrie... (codes 20, quotations 49)	4_Internal Barrier... (codes 20, quotations 57)	5_External Facilit... (codes 23, quotations 113)	6_Internal Facilit... (codes 24, quotations 129)	7_Goals (codes 13, quotations 86)	Totals
2	39	6<br>33.33 %		2<br>11.11 %	8<br>44.44 %	2<br>11.11 %	18<br>100 %
3	55		9<br>31.03 %	16<br>55.17 %	3<br>10.34 %	1<br>3.45 %	29<br>100 %
4	55	1<br>3.57 %	3<br>10.71 %	12<br>42.86 %	8<br>28.57 %	4<br>14.29 %	28<br>100 %
6	37		7<br>36.84 %	5<br>26.32 %	6<br>31.58 %	1<br>5.26 %	19<br>100 %
15	42	7<br>31.82 %		5<br>22.73 %	6<br>27.27 %	4<br>18.18 %	22<br>100 %
17	51	5<br>18.52 %	5<br>18.52 %	4<br>14.81 %	9<br>33.33 %	4<br>14.81 %	27<br>100 %
19	50	2<br>6.45 %	9<br>29.03 %	10<br>32.26 %	4<br>12.90 %	6<br>19.35 %	31<br>100 %
20	48	4<br>13.79 %	3<br>10.34 %	9<br>31.03 %	11<br>37.93 %	2<br>6.90 %	29<br>100 %
21	37	3<br>23.08 %	1<br>7.69 %	2<br>15.38 %	5<br>38.46 %	2<br>15.38 %	13<br>100 %
		28<br>12.96 %	37<br>17.13 %	65<br>30.09 %	60<br>27.78 %	26<br>12.04 %	216<br>100 %

= number of cases = number of codes = number of coded quotations

Cross-Table S3. underlying the Low-Goal Type Cases

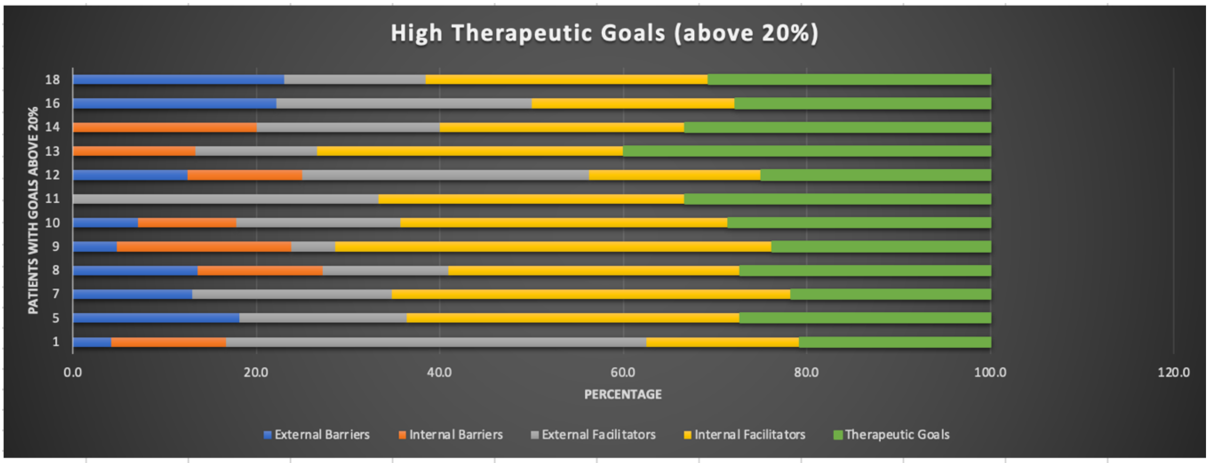

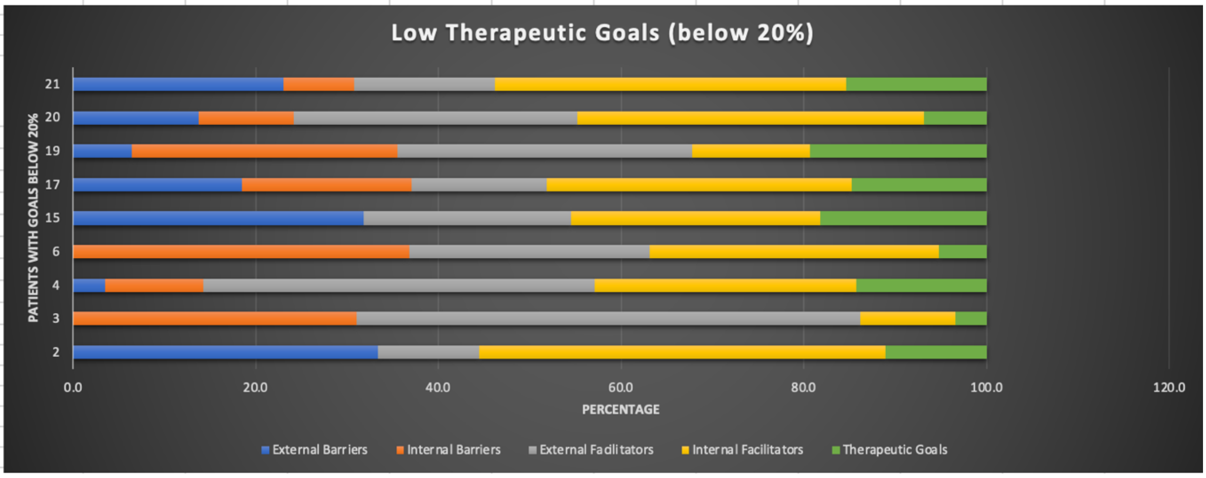

Visualisation S4. Visualised comparison of high-goal-type cases and low-goal-type cases

	5_High Goal Group 12 cases, 449 quotations	5_Low Goal Group 9 cases, 414 quotations	Totals
3_External Barriers to Treatment (20 codes, 49 quotations)	21<br>9.63 %	28<br>12.96 %	49<br>11.29 %
4_Internal Barriers to Treatment (20 codes, 57 quotations)	20<br>9.17 %	37<br>17.13 %	57<br>13.13 %
5_External Facilitators (23 codes, 113 quotations)	48<br>22.02 %	65<br>30.09 %	113<br>26.04 %
6_Internal Facilitators (24 codes, 129 quotations)	69<br>31.65 %	60<br>27.78 %	129<br>29.72 %
7_Goals (13 codes, 86 quotations)	60<br>27.52 %	26<br>12.04 %	86<br>19.82 %
Totals	218<br>100 %	216<br>100 %	434<br>100 %

(document icon) = number of cases (diamond icon) = number of codes (quotation icon) = number of coded quotations

Cross-Table S5. underlying Distribution of Barriers, Facilitators and Therapeutic Goals per Group
